# Investigation of Window Silicone Sealant Weathering Using Evolved Gas Analysis and Pyrolysis Gas Chromatography with Mass Spectrometry

**DOI:** 10.3390/polym17212884

**Published:** 2025-10-29

**Authors:** Eugene Oga, Nafisa Bala, Stephen Fisher, Evguenii Kozliak, Alena Kubátová

**Affiliations:** 1 Department of Chemistry, University of North Dakota, 151 Cornell Street, Grand Forks, ND 58202, USAnafisa.bala@und.edu (N.B.); 2 Marvin Companies, Inc., 802 State Ave, Warroad, MN 56763, USA; stfisher@marvin.com

**Keywords:** silicone sealants, thermal degradation, PDMS pyrolysis, cyclic siloxane speciation, weathering, temperature evolution profiles

## Abstract

Thermal degradation of polydimethylsiloxane (PDMS)-based silicone sealants was investigated using evolved gas analysis (EGA) for rapid temperature screening, combined with multistep pyrolysis gas chromatography with mass spectrometric detection. The identified products were cyclic siloxanes, ranging from hexamethyl cyclotrisiloxane to hexadecamethyl cyclooctasiloxane. Aged and weathered sealants showed distinctly lower siloxane evolution temperatures (450–510 °C) compared to fresh samples (610–710 °C), indicating more facile polymer degradation. This trend was evident in both EGA-MS and Py-GC-MS, with EGA-MS showing a more pronounced effect, suggesting its potential for detecting sealant failure. Notably, the total evolved amounts of specific siloxanes remained nearly constant, suggesting that weathering primarily affects the thermal evolution profiles rather than the overall PDMS structure. The abundance of the three largest-size siloxanes increased (3–7%) with the weathering; in contrast, the changes in the amounts of the most abundant siloxanes were insignificant throughout all samples, regardless of the extent of weathering. These observations suggest that weathering induces changes in details of material structure, e.g., intermolecular interactions, rather than substantial chemical alterations to the PDMS structure.

## 1. Introduction

Industrial sealants serve as a barrier between various materials, preventing leaks and protecting the structural integrity of resulting aggregates. Silicone sealants, e.g., polydimethylsiloxane (PDMS)-based ones, are widely used in window manufacturing due to their strong adhesion, flexibility, and resistance to ultraviolet radiation damage [1,2,3].

However, long-term exposure to UV radiation, high temperatures, ozone, and moisture, i.e., weathering, can result in sealant degradation [4,5]. Current methods to assess the sealant status rely on monitoring mechanical properties [6,7,8] rather than addressing the root of the problem: chemical changes.

Several methods have been employed to identify the specific chemical structure changes resulting from this process. Thermogravimetric analysis (TGA) coupled with FTIR spectroscopy of the evolving products has reported changes in both thermal stability and vibrational frequencies of silicone sealants. Yet, the authors could not identify the underlying chemical processes [7,8,9,10,11,12,13,14]. Gas chromatography (GC) with prior extraction of polymer samples can analyze only the volatile components, as opposed to polymers, so it has found merely limited applications in this problem.

To our knowledge, only one study thus far has been successful in linking specific sealant surface chemistry changes to degradation using FTIR and X-ray photoelectron spectroscopy [15]. Though this study provided valuable information, analysis of the corresponding chemical changes that occur within the bulk of silicone polymers is essential for designing more resilient sealants [3].

Pyrolysis gas chromatography with mass spectrometric detection, Py-GC-MS, is well-suited for detecting chemical changes in complex polymer samples with unknown components [13]. Previous studies on silicone polymer weathering using single-step Py-GC-MS [16,17,18] demonstrated that silicone materials formulated via differing cure chemistries display distinct degradation fingerprints observable by means of analytical pyrolysis. However, they could not differentiate fresh vs. aged/weathered silicone polymers of the same chemistry, nor did they reveal the structural differences between various PDMS-based polymers, presumably due to the limitation of having merely a single pyrolytic temperature step.

We and others have employed an advanced Py-GC-MS system that enables multiple temperature steps for material characterization [19,20,21]. These include thermal desorption (TD), a step conducted near 300 °C, to distinguish volatilized species from pyrolytic products observed in subsequent pyrolytic (Py) steps [19]. This multistep Py-GC-MS method has been successfully applied to complex polymers such as lignin to reveal structural changes resulting from various treatments [20,22].

Earlier studies showed that thermogravimetric analysis (TGA) may be effectively used to obtain the initial temperature profiles of pyrolytic product evolution and the kinetics of thermal degradation of various polymers [23,24]. Instead of TGA, evolved gas analysis (EGA-MS, a similar technique to Py-GC-MS but without chromatographic separation) was successfully used earlier to analyze polymer degradation by recording the volatilized pyrolysis products released as the material heats up [25,26,27].

Our hypothesis tested herein is that the use of multiple temperature steps in Py-GC-MS or a continuous temperature ramp in EGA may reveal the chemical changes occurring in PDMS-based sealants as a result of weathering. The other innovation employed in this work was the use of EGA to obtain qualitative temperature profiles of PDMS sealants, which were then used for assessing the sealants’ status.

To the best of our knowledge, this is the first application of a combined EGA-MS and Py-GC-MS approach to the analysis of silicone sealant weathering. This enhanced method offers the potential to create a unique fingerprint that can be used to identify the material and track its chemical transformations that occur during degradation over time.

## 2. Materials and Methods

### 2.1. Materials

Marvin Companies, Inc. provided for analysis fourteen (14) silicone sealant samples commonly used in residential window applications across the U.S. These samples included two fresh commercial sealants: Sealant 1 and Sealant 2. Twelve samples of Sealant 1, labeled 03 to 14, were subjected to environmental aging through outdoor exposure at residential settings. These samples were exposed to various common weathering and ecological conditions, including UV exposure, high humidity, and significant temperature fluctuations, which are important for evaluating the weathering effects on the sealant’s properties.

In these experiments, approximately 150–200 µg of each sample was introduced using deactivated stainless-steel Eco cups supplied by Frontier Laboratories Ltd (Frontiers Lab, Fukushima, Japan). These cups, with an inner diameter of 3.8 mm, a height of 8 mm, and a volume of 80 µL, were cleaned before use. This cleaning process involved a two-step procedure: first, sonication in acetone, followed by heating to a red-hot state using a butane torch for 3 s. The cleaned cups were then cooled and stored in a desiccator, ready for sample introduction. The specific weather levels of these samples, as determined by EGA-MS and presented in Table 1, will be discussed in detail under Results and Discussion.

### 2.2. Methods

All experiments were conducted using a Frontier Laboratories PY-3030D multi-shot pyrolyzer with an AS-1020E Lab autosampler (Frontiers Lab, Fukushima, Japan) for EGA-MS and Py-GC-MS analyses. This system was coupled to an Agilent Technologies 7890A GC with a 5975C inert XL MS detector equipped with an electron ionization source (Agilent, Santa Clara, CA, USA). Although not used in the analysis, a disconnected Frontier Laboratory Selective Sampler SS-1010E was attached to the system, with a sampling time of 10 s.

In EGA-MS mode, a deactivated tube replaced the GC column to enable the direct transfer of evolved gases from the pyrolyzer to the mass spectrometer. The total ion current (TIC) mode was used.

Based on the thermal degradation patterns observed in the EGA-MS analyses, a sequential temperature step approach was designed for TD followed by Py, each followed by a GC-MS analysis to differentiate samples according to their weathering and to target specific stages of polymer breakdown. A cryotrap with a temperature range of −190 °C to 350 °C was used. The cryotrap served to concentrate the desorbed volatile compounds, preventing them from immediately entering the GC column. This focusing step was crucial for improving the analysis sensitivity and resolution, allowing for the detection of volatile components that might otherwise be lost.

This approach allowed for a detailed comparative analysis of fresh and aged sealant samples. To ensure the effectiveness of the Py-GC-MS method, the heating rate was evaluated as described in the corresponding section of Results and Discussion. The final pyrolyzer temperature program, as well as the GC and MS conditions for both modes, are listed in Table 2.

### 2.3. Data Processing

The siloxane product identification was performed using the NIST 20 library with the percentage match reported. Semi-quantification was performed only for the Py-GC-MS data. Based on the assumption that the ionization efficiency and response factors of cyclic siloxanes are similar, semi-quantification of each product was conducted by measuring the integrated area of its corresponding total ion current (TIC) peak in the chromatogram.

Since the analytes are of similar chemical nature, the peak area can be assumed to be proportional to the relative amount of that product present. Therefore, the results were reported as normalized areas of TIC, where the fraction of each product (siloxane) was calculated by dividing its area by the total area of all six siloxane peaks. This procedure provided a relative abundance for each product within each temperature step.

To determine whether significant differences existed among the thermal groupings, i.e., Level 1–Level 3, a one-way analysis of variance (ANOVA) was conducted. Data analysis was performed using two statistical software packages: Minitab 18 (Minitab LLC, State College, PA, USA) and JMP 18 (SAS Institute Inc., Cary, NC, USA). The level of significance was set at α = 0.05. The *p*-value represents the probability of determining whether there is a significant difference between the obtained mean values.

## 3. Results and Discussion

### 3.1. Characterization of Sealants Using EGA-MS

**Figure 1:**
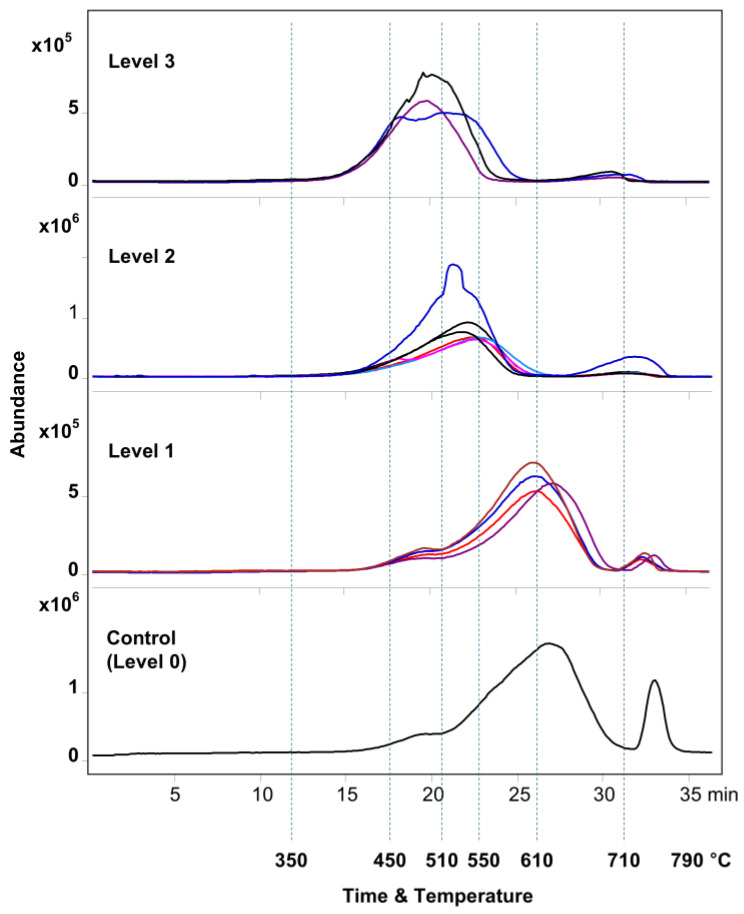
EGA-MS thermograms of silicone sealant samples were divided into the level groups (Level 3, Level 2, Level 1, and Level 0) based on their temperature profiles associated with the extent of weathering. The vertical dashed lines denote the temperature ranges used subsequently for Py-GC-MS.

The initial temperature screening of various sealants using EGA-MS (Figure 1) provided insight into their thermal degradation behavior resulting from weathering. As one can see, the evolution temperatures of pyrolytic products varied between fresh and weathered sealants. Based on the magnitude of this temperature shift to lower values compared to the freshly prepared commercial sealant used as the control (i.e., zero level of degradation, Level 0), the analyzed real-world samples were grouped into three levels, 1 through 3, going from the minimum to maximum exposure or level of weathering. These are labeled henceforth as Level 1, Level 2, and Level 3, respectively. For the Py-GC-MS experiments, one representative sample was selected from each group. The samples chosen were #13 from the L1 group, #09 from the L2 group, and #5 from the L3 group.

The positions of both the first, major peak (reflecting a partial breakdown of PDMS to cyclic siloxanes) and the second, smaller peak (complete PDMS breakdown) shifted toward lower evolution temperatures with weathering. These shifts indicate that polymer degradation occurs more readily in samples with more prolonged environmental exposure. Therefore, the change in the temperature corresponding to the maximum of the first EGA-MS peak can be related to the extent of weathering.

The broad major peak in EGA-MS thermograms (eluting between 20 and 26 min, i.e., 500 and 620 °C for L3 and L0, respectively) of all samples (Figure 1) suggested the release of cyclic siloxanes (confirmed later by Py-GC-MS, see Table 3 and Figure S1b), products of PDMS pyrolysis, as shown by the use of extracted ion thermograms and mass spectra (Figure S1a). The second, minor peak (eluting between 30 and 33 min for L3 and L0, respectively), evolving at higher temperatures, corresponds to carbon dioxide (CO_2_), as evidenced by the characteristic ion at 44 *m*/*z* and the supporting mass spectrum (Figure S1a). Silicone sealants are complex mixtures containing various additives, fillers, and cross-linking agents. The CO_2_ peak occurrence is a direct result of the thermal decomposition of these non-siloxane components [28,29]. For example, common fillers like calcium carbonate (CaCO_3_) will decompose under the high temperatures of pyrolysis to form calcium oxide (CaO) and CO_2_ [30]. Therefore, the presence and size of this peak provide a clear indication of the quantity and type of these thermally decomposable oxygen-containing components within the sealant, confirming that the CO_2_ is a key pyrolyzate rather than an air contaminant. This peak may also indicate the breakdown of oxygen-containing functional groups within the polymer; a process associated with increased thermal decomposition.

The next section details the chromatographic separation and distribution of the observed siloxanes among the various temperature steps, further elucidating the thermal degradation patterns observed in the samples.

### 3.2. Distribution of Siloxanes Among the Sequential Pyrolyzer Temperature Steps Determined with Py-GC-MS

To further investigate the changes in sealant thermal degradation profiles due to weathering, Py-GC-MS was employed. An essential parameter in multistep Py-GC-MS is the heating rate, which influences product separation and signal quality. Therefore, two heating rates were compared: 20 °C/min (the standard rate) and 600 °C/min (fast), with the results shown in Figure S2 for the freshly prepared control sample. While the presumed standard rate (20 °C/min) and the faster rate (600 °C/min) provided similar product separation, the rapid heating at 600 °C/min did not allow for complete product evolution at the designated temperature steps, hindering accurate siloxane semi-quantification. Consequently, a heating rate of 20 °C/min was selected for subsequent experiments. This choice was validated by successfully differentiating two distinct commercial sealants (Figure 6a).

**Figure 2:**
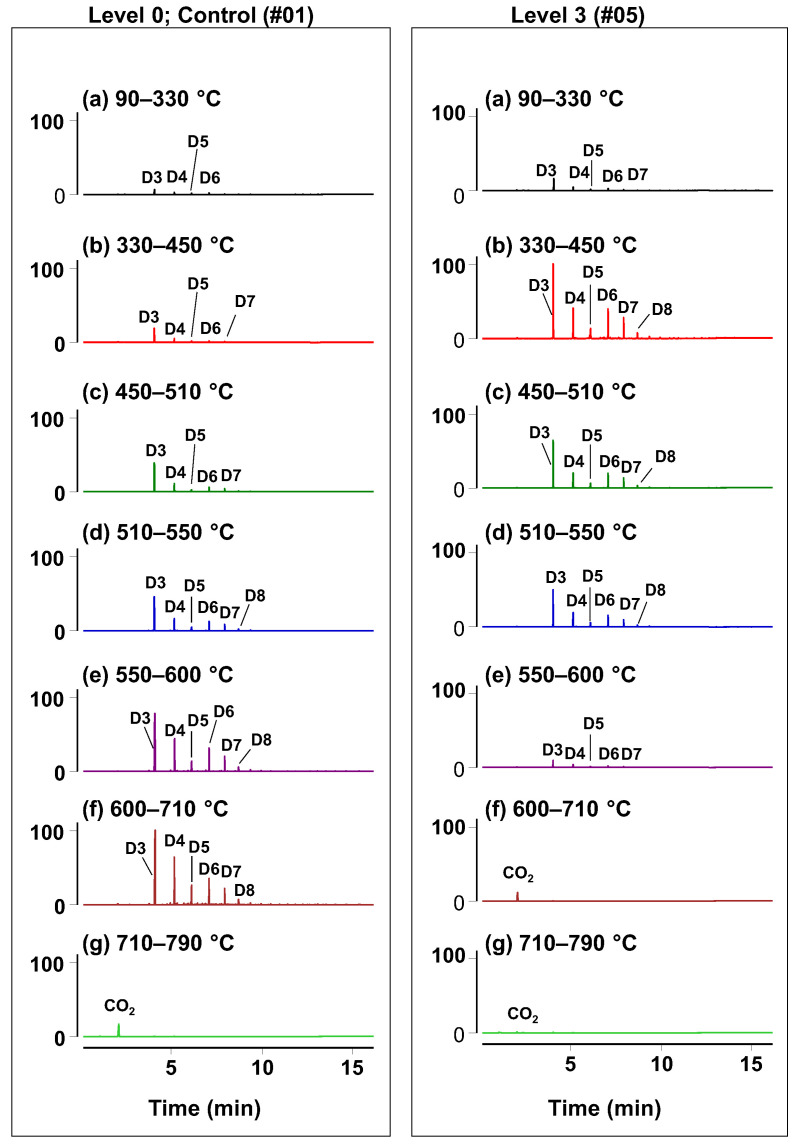
Py-GC-MS analysis of control (Level 0, #01) and weathered (Level 3, #04) sealants. Total ion current (TIC) chromatograms (**a**–**g**) were obtained sequentially using seven pyrolyzer temperature steps, as outlined in Figure 1 and Table 2. Peak labels: D3 through D8 correspond to cyclic siloxanes, respectively, as detailed in Table 3. The (**a**–**g**) of chromatograms reflects the discrete temperature steps indicated, not repeated measurements. The adopted labeling is based on that previously reported [31] with the number representing monomer units in a given oligomer.

With the established Py-GC-MS conditions for fresh sealants, the focus shifted to examining the samples with varying degrees of weathering. This is demonstrated in Figure 2, which shows representative chromatograms for the control sample (Level 0, #01) and a significantly aged sample (Level 3, #04). The complete set of chromatograms for all samples is presented in Figure S3a–c. The observed distinctive differences in the Py-GC-MS profiles obtained with sequential temperature steps enabled a detailed investigation of sealant changes that occurred due to weathering.

The formulas and structures of the identified cyclic siloxanes identified using the Py-GC-MS are listed in Table 3, along with their corresponding retention times, identification, and confirmation ions. This table provides a comprehensive overview of the siloxane species detected across the different pyrolyzer temperature steps, which were designed based on the EGA-MS findings to differentiate samples according to their weathering.

**Figure 3:**
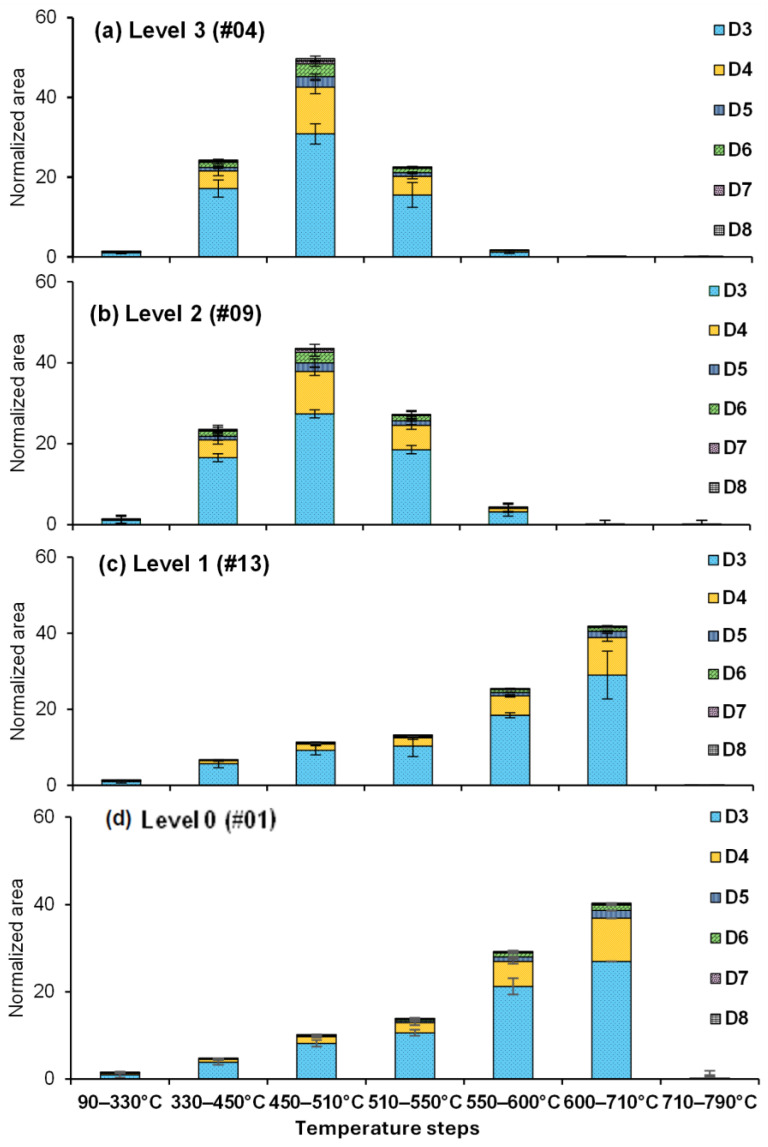
Siloxane distribution across pyrolyzer temperature steps in samples of varied levels of degradation: (**a**) Level 3 (#04), (**b**) Level 2 (#09), (**c**) Level 1 (#13), and (**d**) Level 0 (#01) (see Figure S3 for all samples). The data are presented as mean values of normalized peak areas (% total siloxane, per µg sample) for each temperature step, with one standard deviation (*n* = 3) as the error bar.

Building on the initial grouping of sealants based on their EGA-MS thermograms (cf. Figure 1), the trends in the abundance of identified siloxanes observed using Py-GC-MS analysis were investigated for varied levels of exposure. The corresponding data are shown for representative samples in Figure 3 (the remaining samples are shown in Figure S4).

While the overall evolution pattern of the major thermal degradation products, cyclic siloxanes (Figure 2), observed for freshly prepared sealants, was similar to what had been reported in previous studies [32], we also noted the distinct differences in thermal degradation profiles (Figure 3). A similarity was observed in the identification of the dominant peak as D3, followed by D4–D8, showing a pattern of gradual decline in peak size with increasing molecular mass. This observation was explained by the preferred formation of a thermodynamically favored 6-membered cyclic compound [33,34].

**Figure 4:**
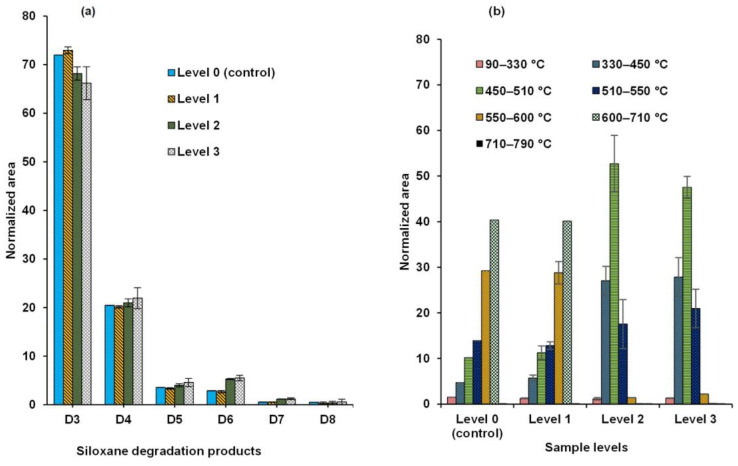
Cumulative cyclic siloxane amounts in control and weathered samples. (**a**) Cumulative amounts of each siloxane (D3–D8) across all temperature steps are normalized to 100%. (**b**) Siloxane distribution across TD-Py temperature steps within each level of weathering, normalized to 100% area composition, is presented as mean values with one standard deviation (*n* = 3).

Consistent with this pattern, the Py-GC-MS analysis nonetheless revealed distinctly different thermal degradation temperature profiles for aged/weathered sealant samples (Figure 2, Figure 3, Figure 4 and Figures S3 and S4). This is a novel finding compared to any prior work, enabled by the use of multiple temperature steps in Py-GC-MS. For Level 3, most of the pyrolytic products evolved within a specific temperature range of 300–550 °C (Figure 3a), indicating a more facile degradation compared to fresh samples (Level 0). In contrast, Level 2 sealants exhibited a slightly wider evolution temperature range compared to Level 3, reaching up to 600 °C (Figure 3b).

In contrast, both Level 1 and fresh samples demonstrated continuous degradation up to 710 °C, with a significant increase starting at 510 °C (Figure 3c,d). This difference suggests that the thermal degradation of fresh and near-fresh sealants is hindered, occurring at higher temperatures and over a broader range. These findings underscore the variability in degradation patterns observed even among sealants of the same age, as evidenced by the distinct profiles of certain Level 3 and Level 2 samples despite their similar age (Figure 3 and Figure S4).

Confirming this observation, sealants of varying ages within the same weathering group exhibited similar thermal elution patterns. For instance, samples exposed for 11, 12 and 13 years (all within one weathering group, Level 3) exhibited similar evolution temperatures, as depicted in Figure S3a,b. Similarly, samples from 1, 3, and 8 years (weathering group Level 1) also displayed consistent elution profiles, as shown in Figure S3c. Despite variations in age, samples of the same level showed similar weathering, indicating that the degree of material degradation, rather than the duration of environmental exposure, is the primary determinant of the observed thermal evolution patterns of cyclic siloxanes.

This finding suggests that the cumulative impact of weathering on the sealant structure has a direct influence on its thermal degradation behavior. This observation is consistent with the earlier conclusion that factors such as environmental exposure, chemical interactions, and manufacturing inconsistencies can contribute to the variability in thermal stability and degradation mechanisms of silicone sealants [33].

Longer-exposed/weathered sealants exhibited a differing temperature range, i.e., the D3–D8 cyclic siloxanes completely evolved at lower temperatures compared to fresh samples. The evolution of degradation products occurred within defined temperature ranges, providing a means to determine whether a particular sealant has been significantly weathered. Due to the use of varied temperatures in Py-GC-MS in this work, this feature became observable, unlike previous studies that employed single-temperature pyrolysis-gas chromatography [18] or two-dimensional GC-MS-TOF [17] approaches with principal component analysis. Namely, the lack of temperature variation may explain the lack of difference in the siloxane evolution pattern between fresh and weathered samples reported by Lewicki et al. [17].

The trends observed in Py-GC-MS are qualitatively similar to those obtained through EGA-MS, mutually validating both techniques. However, unlike fresh or slightly aged samples, for which the EGA-MS and Py-GC-MS results were rather similar, Py-GC-MS applied to significantly weathered samples showed a slightly greater evolution of pyrolytic products at lower temperatures compared to EGA-MS, with a corresponding decrease in higher-temperature fractions.

The difference between the EGA and Py-GC-MS setups presumably lies in the short time intervals between the temperature ramping steps in the Py-GC-MS setup. During these intervals, the samples are temporarily removed from the heating zone, although not immediately. The resulting extended residence time during all temperature steps in Py-GC-MS may have contributed to the partial evolution of pyrolytic products at lower temperatures compared to EGA-MS.

The combined application of EGA-MS and Py-GC-MS yields a more comprehensive understanding of the thermal degradation of weathered silicone sealants. The former provides a quick qualitative method for determining the sealant status, whereas the latter enables accurate semi-quantification, which is essential for statistical analysis considered in the next section.

### 3.3. Weathering with Regard to Siloxane Speciation

Multiple cases of altered thermally evolved siloxane speciation upon PDMS breakdown have been reported in the literature, indicating changes in chemical composition. For example, a significant increase in the high-MW siloxane fraction, namely D5 to D8 at the expense of D3, was observed by Li et al. when PDMS was co-polymerized with methyl methacrylate [35]. A similar alteration of the cyclic siloxane thermal evolution pattern was observed by Lewicki et al. in PDMS composites with nano clay, montmorillonite [36].

In another study by the same group, the influence of crosslinking on siloxane speciation was investigated. Short crosslinkers promoted the formation of D4 to D5, i.e., smaller-MW siloxanes, whereas the evolution of higher-MW products, even beyond D8, was observed with long crosslinkers [31]. These results were consistent with those observed by Maxwell et al. and Chinn et al., who studied the radiative PDMS weathering and observed that radiation-induced crosslinking caused an increase in the MW of the evolved siloxanes [37,38].

Therefore, we set out to investigate whether the evolved pyrolytic siloxane speciation depends on the weathering of the sealant. We observed that the overall composition of siloxanes from the same PDMS base remains virtually the same (Figure 4a). However, at the same time, our data showed some minor differences in siloxane occurrence caused by weathering.

Namely, the data presented in Figure 4a show that the total siloxane composition was consistent for all representative samples. Specifically, the sums of D3 and D4 were similar in both fresh and significantly aged sealants. The amounts of D6 and D8 slightly increased with weathering, while that of D5 decreased. These differences, accumulated with weathering, were qualitatively similar to those reported in the literature, although the magnitude of these effects was much smaller.

The consistency of each siloxane’s composition was confirmed across all samples. Figure S5 presents the sum of all siloxane products per sample: (a) Levels 1 vs. control, (b) Levels 2 vs. control, and (c) Levels 3 vs. control.

Nonetheless, the temperature profiles of weathered samples, Levels 2 (#09) & 3 (#05), were significantly different from less aged (Level 1) and fresh (Level 0) samples, as shown in Figure 4b. That is, while the overall evolving siloxane composition remains constant (or nearly constant), their thermal evolution temperature profile is significantly affected by weathering.

**Figure 5:**
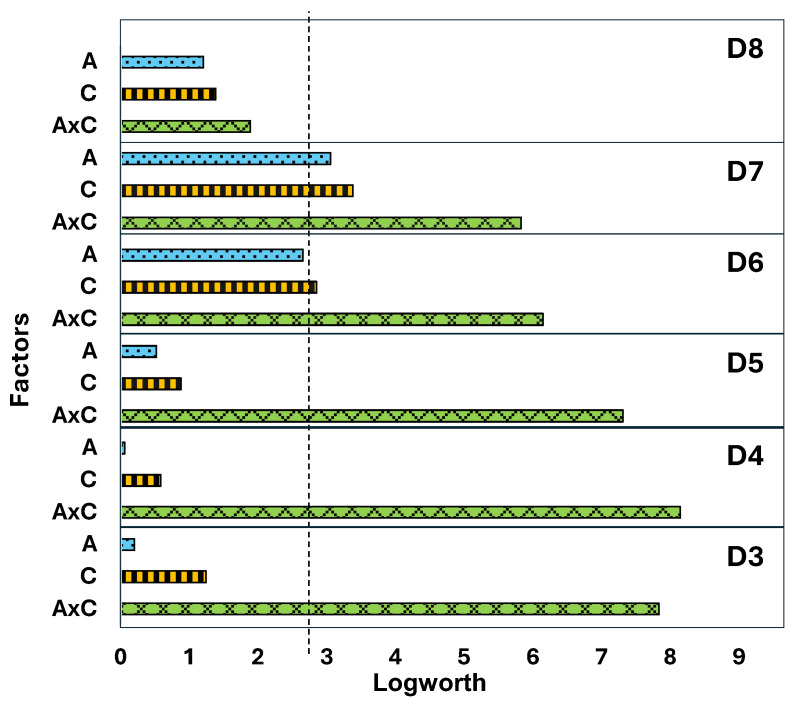
Statistical analysis of factors affecting the pyrolytic evolution of cyclic siloxanes. Factor A represents sample age, factor C represents evolution temperature, and A × C denotes their interaction. The dashed vertical line indicates the *p*-value threshold of 0.05.

To determine which of these effects were statistically significant, the obtained data set was subjected to an ANOVA analysis, as presented in Figure 5. The corresponding linear regression analysis data are shown in Table S1.

The statistical analysis conducted investigated the effects of sample age (Factor A), pyrolysis temperature (Factor C), and their interaction (A × C) on the outcome variable, siloxane composition. The statistical treatment confirmed that neither the temperature itself nor sample age per se (i.e., the sum of siloxane peaks of a certain type across all temperatures for a given sample, as in Figure 4a) was statistically significant, *p*-value < 0.05. The slight increase in the D6 and D7 sums, as shown in Figure 4a, was statistically significant, although barely so for D6, as could be seen in Figure 5, *p*-values shown in Table S1.

For the most abundant products, D3–D8, the corresponding changes were not statistically significant, despite any differences in environmental exposure. In contrast, the interaction of temperature and age, i.e., thermal evolution profiles as a function of weathering, was shown to have high statistical significance.

Linear regression analysis was conducted to model the relationship between the outcome variable and the factors (A, C, and A × C). The analysis provided estimates of the coefficients for each factor, indicating the strength and direction of their influence on the outcome. The significant interaction term (A × C) indicates that the combined effect of sample age and temperature on the outcome is not simply additive. The impact of one factor may be moderate or amplified by the level of the other factor.

**Figure 6:**
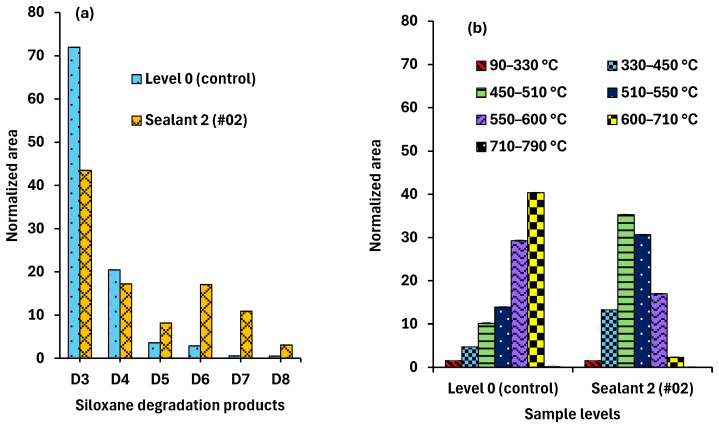
Py-GC-MS results for a different PDMS Sealant 2 compared to Sealant 1 used as the control for aged samples (Level 0). (**a**) Total evolved siloxanes and (**b**) Siloxane distribution across the temperature steps.

Thus, the observed changes resulting from sealant weathering are unlikely to be explained by alterations in the PDMS structure. This finding, combined with the alteration in evolution temperature profiles, may suggest changes in material texture or intermolecular interactions as a result of weathering. To test this possibility, a different fresh PDMS sealant (Sealant 2, #02, see Table 1) was analyzed, and the obtained samples were subjected to the same Py-GC-MS protocol. The results are shown in Figure 6 in comparison to Level 0, i.e., the control fresh sealant, with panels a and b presenting similar information to the corresponding panels of Figure 4.

The temperature profiles obtained turned out to be rather different for the two fresh sealants, despite the similar PDMS-based structure and lack of weathering. For the other fresh PDMS-based sealant, #2 (Table 1), both the total amounts of certain siloxanes across all temperatures (Figure 6a) and the siloxane speciation at certain temperatures (Figure 6b) resembled the weathered samples of the first sealant (groups Level 2–Level 3) much more than the corresponding control sample, Level 0. The observation that a structurally similar PDMS sealant having different other components displayed a pyrolytic profile resembling aged/weathered samples underscores the significant influence of minor components, either accumulated due to environmental exposure or introduced during manufacturing.

The incorporation of manufacturing additives in silicone sealants, such as reinforcing fillers and plasticizers, significantly alters the intermolecular interactions within the PDMS matrix, thereby influencing its thermal degradation behavior. For example, reinforcing fillers, such as fumed silica, create a physical “cross-link” network by forming hydrogen bonds with the PDMS polymer chains. This network restricts chain movement, making it more difficult for the sealant to undergo thermal decomposition and altering the degradation pathway [31].

Conversely, plasticizers, which are typically low-MW silicone fluids, insert themselves between the PDMS chains, increasing the free volume and weakening the intermolecular forces. This makes it easier for the polymer to break down, thereby lowering the overall decomposition temperature and modifying the degradation products [39]. This phenomenon parallels the impact of weathering-induced chemical changes, as both scenarios disrupt the inherent intermolecular forces within the sealant, leading to modifications in its thermal degradation properties.

In addition, the sensitivity and precision of the analytical techniques employed can also contribute to the observed variations. These findings underscore the critical importance of including multiple control samples in any study to account for inherent material variations and experimental uncertainties, thus enabling accurate statistical analysis.

## 4. Conclusions

Aged and weathered PDMS-based silicone sealants exhibited distinctly different thermal degradation profiles compared to the corresponding fresh samples. The evolution of pyrolytic products occurred at lower temperatures in environmentally exposed sealants, with further shifts to even lower temperatures occurring with increased weathering. This finding confirms the hypothesis that thermal evolution methods can be applied to these materials. Contrary to initial expectations, however, the semi-quantitative Py-GC-MS analysis revealed merely minor statistically significant changes in the overall product speciation evolved due to weathering.

Therefore, sealant weathering appears to primarily affect the interactions of silicone polymer molecules with each other and with minor sealant components, rather than the PDMS structure. This loss of crosslinking lowers the energy required for thermal degradation, causing the evolution of products at lower temperatures.

Both EGA-MS and its combination with multistep Py-GC-MS can thus be used to determine the extent of silicone sealant weathering. While EGA-MS is less expensive, faster, and more effective in capturing the extent of weathering, Py-GC-MS can provide more detailed information on subtle differences in degradation behavior and siloxane speciation, which may be influenced by the sample’s history and processing conditions.

## Figures and Tables

**Table 1:** Description of sealant samples used in analysis.

Sample #	Sample Description: Number of Years Exposed	Weather Level Based on EGA-MS
Fresh (unweathered) sealant		
01	Sealant 1 (Control)	L0
02	Sealant 2 *	NA **
Aged samples compared with Sealant 1 (Control L0)		
03	13 years	L2
04	12 years	L3
05	12 years	L3
06	12 years	L3
07	12 years	L2
08	11 years	L2
09	11 years	L2
10	9 years	L2
11	8 years	L1
12	3 years	L1
13	1 year	L1
14	1 year	L1

* Sealant 2 was a different fresh PDMS sealant to be compared only with the pertinent fresh sealant sample (Sealant 1, i.e., Control L0). Therefore, Sealant 2 is not to be compared with aged/weathered samples. ** NA denotes “not applicable.”

**Table 2:** Experimental EGA-MS and Py-GC-MS conditions.

Parameter	EGA-MS Mode	Py-GC-MS Mode
Carrier gas	Helium, 1.0 mL/min	Helium, 1.0 mL/min
Pyrolyzer temperature	90–800 °C (ramp 20 °C/min, hold 1 min)	7 steps (90–330 °C, 330–450 °C, 450–510 °C, 510–550 °C, 550–600 °C, 600–710 °C, 710–790 °C) at 20 °C/min
Injector	300 °C, split 1:100	300 °C, split 1:100
Cryogenic trapping	NA	Liquid nitrogen
GC oven temperature program	300 °C	40–300 °C, ramp 25 °C/min, hold 4 min
Column	Ultra ALLOY-DTM deactivated tube, 0.15 mm ID × 2.5 m, (Frontier Laboratories)	DB-5MS capillary column, 40 m × 250 μm ID × 0.25 µm film thickness (J&W Scientific, Folsom, CA, USA)
Transfer line temperature	300 °C	300 °C
Ionization source	EI (70 eV), 230 °C	EI (70 eV), 230 °C
MS Quad	150 °C	150 °C
MS scan range	10–700 *m*/*z*	10–700 *m*/*z*
Scan rate	scan/6 s	scan/2.66 s

**Table 3:** Identified cyclic siloxane characteristics, including their labeling, formulas, retention times (t_R_), and confirmation ions (*m*/*z*) observed in Py-GC-MS. The structures are shown in Figure S1b.

Name	Hexamethylcyclo-trisiloxane	Octamethylcyclo-tetrasiloxane	Decamethylcyclo-pentasiloxane	Dodecamethylcyclo-hexasiloxane	Tetradecamethylcyclo-heptasiloxane	Hexadecamethylcyclooctasiloxane
Label *	D3	D4	D5	D6	D7	D8
Formula	C_6_H_18_O_3_Si_3_	C_8_H_24_O_4_Si_4_	C_10_H_30_O_5_Si_5_	C_12_H_36_O_6_Si_6_	C_14_H_42_O_7_Si_7_	C_16_H_18_O_8_Si_8_
t_R_ (min)	6.07	7.77	8.64	9.56	10.39	10.90
*m*/*z*	207	281	355	429	503	577

* This label (e.g., D3) is given for reference, as it is often used in the literature [31], representing the number of monomer units in a given oligomer.

## Data Availability

The original contributions presented in this study are included in the article and Supplementary Materials. Further inquiries can be directed to the corresponding authors.

## Supplementary Materials

The supporting information can be downloaded at: https://www.mdpi.com/article/10.3390/polym17212884/s1, Investigation of Window Silicone Sealant Weathering Using Pyrolysis Gas Chromatography and Mass Spectrometry. Figure S1a: The EGA-MS data (a). The EGA-MS thermograms of all samples, (b) Total ion and extracted ion thermograms (suggesting release of cyclic siloxanes, products of PDMS pyrolysis). (c) MS spectra for the two peaks in the thermogram. The second minor peak, evolving at higher temperatures, corresponds to carbon dioxide (CO_2_), as evidenced by the characteristic ion at 44 *m*/*z*; Figure S1b: Structures and MS spectra of identified siloxane products with their retention times; Figure S2: Py-GC-MS chromatograms for the freshly prepared control (L0) sample comparing two heating rates used for pyrolysis experiments. Panes a-e represent chromatograms obtained with sequential temperature steps: (a) 90–390 °C, (b) 390–530 °C, (c) 530–630 °C, (d) 630–690 °C, (e) 690–790 °C; Figure S3: Py-GC-MS chromatograms for the remaining samples (a) for L3 samples, (b) for L2 samples and (c) L1 samples; Figure S4: The semi-quantitative Py-GC-MS analysis obtained in 7 sequential Py steps: obtained with sequential temperature steps: 90–330 °C, 330–450 °C, 450–510 °C, 510–550 °C, 550–600 °C, 600–710 °C, 710–790 °C. Highlighted is the representative sample shown in Figure 3. (a) Level 3, (b) Level 2, and (c) Level 1; Figure S5: Sum of all siloxane products (TIC peak areas) per sample. This reveals that the overall composition of the siloxanes from the same PDMS base is equal, but a shift to lower Temperatures is observed in Figure S4. The comparison of individual samples is shown below. (a) Level 1 (L1) vs. control, (b) Level 2(L2) vs. control, (c) Level 3 (L3) vs. control. Table S1. Details of linear regression analysis.

## Abbreviations

The following abbreviations are used in this manuscript:

Py-GC-MS	Pyrolysis gas chromatography mass spectrometry
EGA-MS	Evolved gas analysis and mass spectrometry
PDMS	Polydimethylsiloxane
TGA	Thermogravimetric analysis
TIC	Total ion current
ANOVA	Analysis of variance
