# Supplementary material for: Investigation of Window Silicone Sealant Weathering Using Evolved Gas Analysis and Pyrolysis Gas Chromatography with Mass Spectrometry
Source: Polymers (Basel). 2025 Oct 29;17(21):2884. doi: 10.3390/polym17212884 (PMC12610109; doi:10.3390/polym17212884)
Supplement: Supplementary file 1 [file polymers-17-02884-s001.zip › polymers-3929548-supplementary.pptx]

## Slide 1
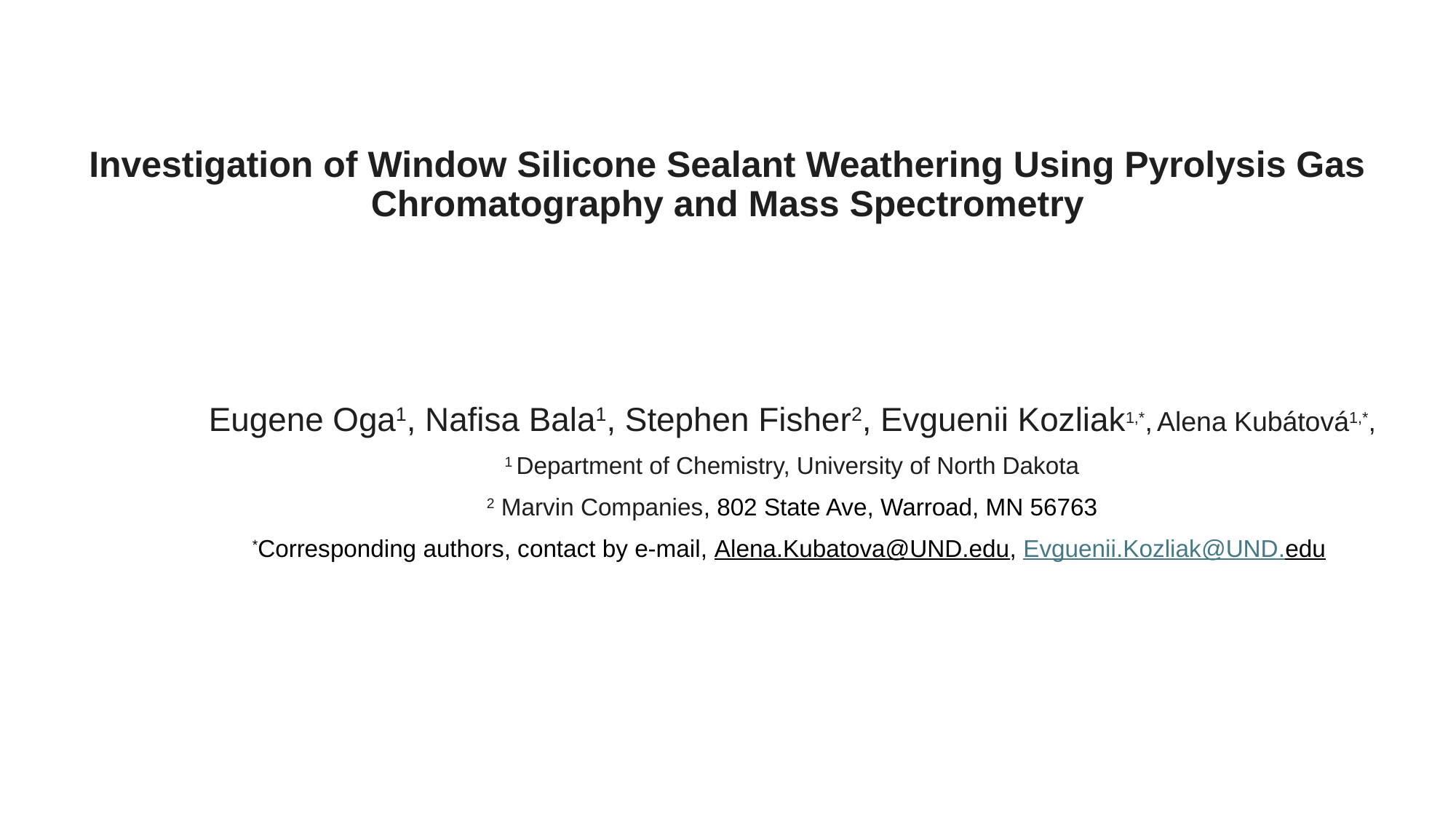

# Investigation of Window Silicone Sealant Weathering Using Pyrolysis Gas Chromatography and Mass Spectrometry
Eugene Oga1, Nafisa Bala1, Stephen Fisher2, Evguenii Kozliak1,*, Alena Kubátová1,*,
1 Department of Chemistry, University of North Dakota
2 Marvin Companies, 802 State Ave, Warroad, MN 56763
*Corresponding authors, contact by e-mail, Alena.Kubatova@UND.edu, Evguenii.Kozliak@UND.edu

## Slide 2
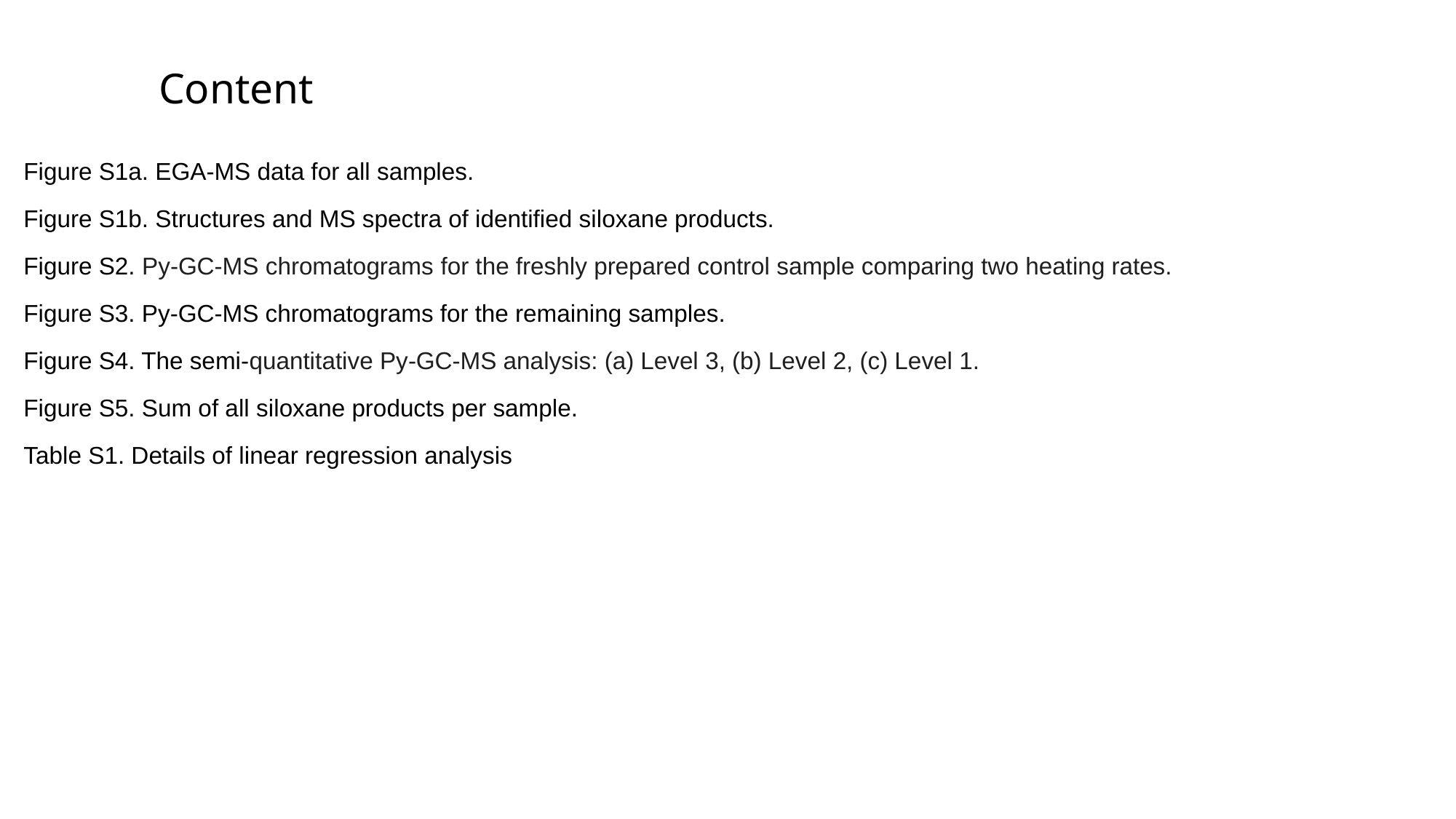

# Content
Figure S1a. EGA-MS data for all samples.
Figure S1b. Structures and MS spectra of identified siloxane products.
Figure S2. Py-GC-MS chromatograms for the freshly prepared control sample comparing two heating rates.
Figure S3. Py-GC-MS chromatograms for the remaining samples.
Figure S4. The semi-quantitative Py-GC-MS analysis: (a) Level 3, (b) Level 2, (c) Level 1.
Figure S5. Sum of all siloxane products per sample.
Table S1. Details of linear regression analysis

## Slide 3
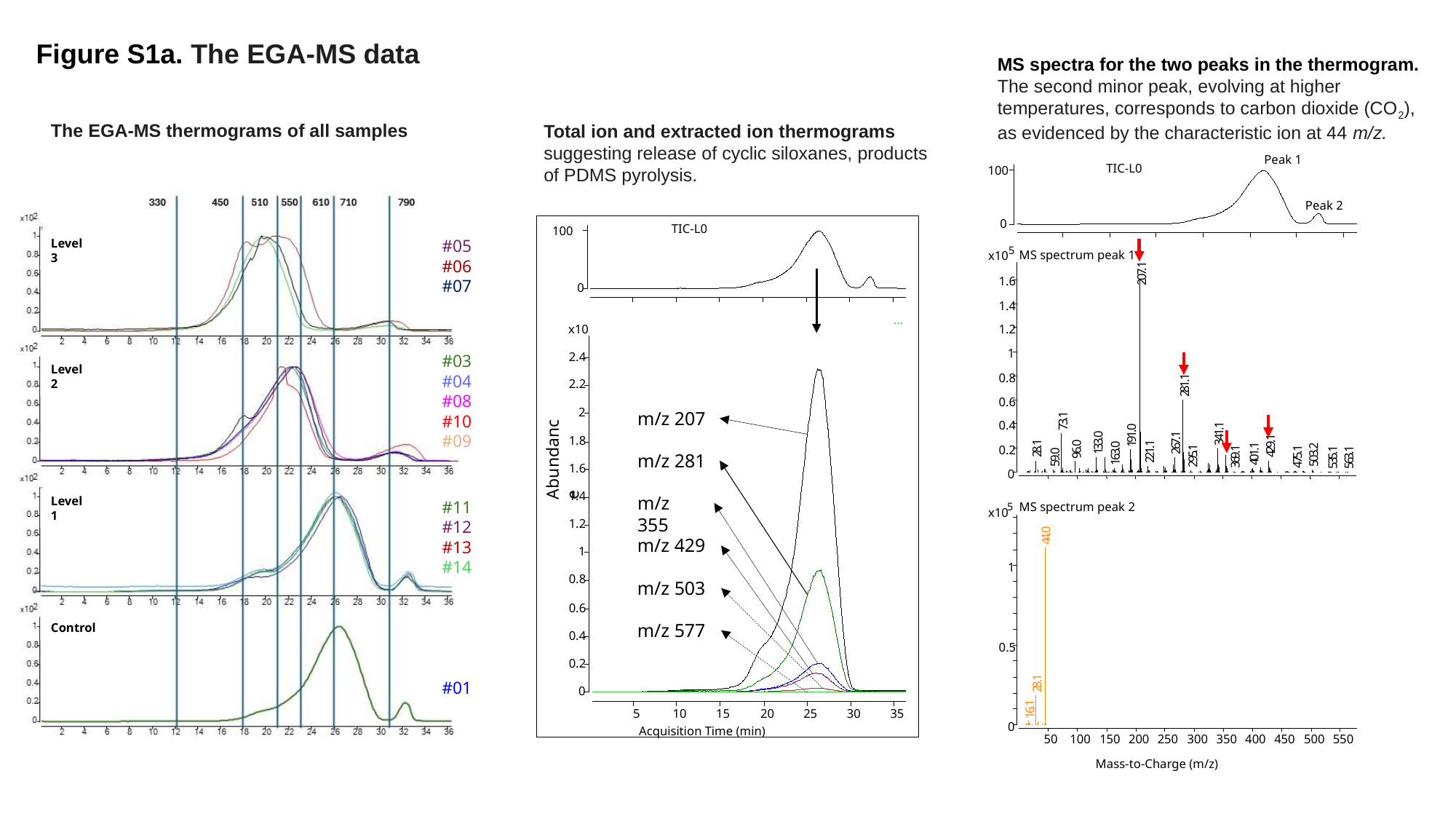

# Figure S1a. The EGA-MS data
MS spectra for the two peaks in the thermogram.
The second minor peak, evolving at higher temperatures, corresponds to carbon dioxide (CO2), as evidenced by the characteristic ion at 44 m/z.
The EGA-MS thermograms of all samples
Total ion and extracted ion thermograms
suggesting release of cyclic siloxanes, products of PDMS pyrolysis.
Peak 1
TIC-L0
100
0
5
MS spectrum peak 1
x10
1.6
1.4
1.2
1
0.8
0.6
0.4
0.2
0
1
.
7
0
2
1
.
1
8
2
1
.
3
1
7
0
.
.
1
0
1
1
4
1
.
.
9
7
.
3
1
3
0
1
1
9
0
1
.
6
2
3
1
.
.
1
1
1
1
.
8
.
2
.
6
1
2
.
0
1
.
.
3
1
.
.
3
2
5
4
9
5
.
9
2
5
3
6
0
0
9
9
2
6
7
3
6
1
4
5
5
2
3
4
5
5
MS spectrum peak 2
5
x10
0
.
4
4
1
0.5
1
.
8
2
1
.
6
1
0
50
100
150
200
250
300
350
400
450
500
550
Mass-to-Charge (m/z)
Peak 2
Level 3
#05
#06
#07
#03
#04
#08
#10
#09
Level 2
Level 1
#11
#12
#13
#14
Control
#01
TIC-L0
100
0
…
x10
2.4
2.2
2
1.8
1.6
1.4
1.2
1
0.8
0.6
0.4
0.2
0
5
10
15
20
25
30
35
Acquisition Time (min)
m/z 207
Abundance
m/z 281
m/z 355
m/z 429
m/z 503
m/z 577

## Slide 4
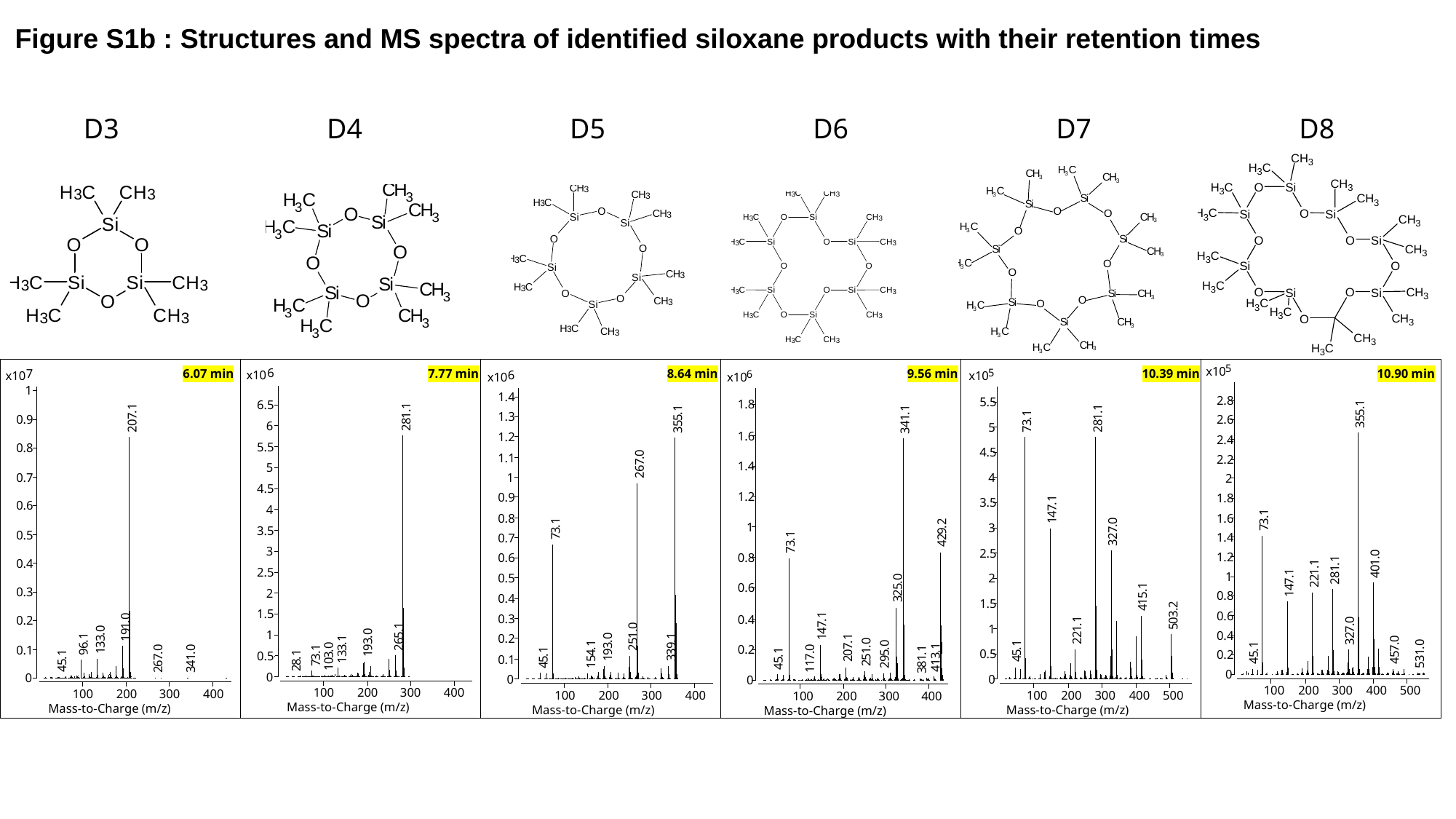

# Figure S1b : Structures and MS spectra of identified siloxane products with their retention times
D3
D4
D5
D6
D7
D8
5
x10
2.8
2.6
2.4
2.2
2
1.8
1.6
1.4
1.2
1
0.8
0.6
0.4
0.2
0
1
.
5
5
3
1
.
3
7
0
.
1
1
1
.
.
1
0
1
1
8
4
.
2
7
2
2
4
1
0
.
7
2
0
3
0
.
1
7
.
.
1
5
5
3
4
4
5
100
200
300
400
500
Mass-to-Charge (m/z)
6
x10
1.8
1.6
1.4
1.2
1
0.8
0.6
0.4
0.2
0
1
.
1
4
3
2
.
9
1
2
.
4
3
7
0
.
5
2
3
1
.
7
4
1
1
0
.
0
7
.
1
0
.
1
1
1
.
.
0
5
.
.
3
7
5
1
2
9
5
1
1
2
8
2
4
4
1
3
100
200
300
400
Mass-to-Charge (m/z)
5
x10
5.5
5
4.5
4
3.5
3
2.5
2
1.5
1
0.5
0
1
.
1
1
.
3
8
7
2
1
.
7
4
1
0
.
7
2
3
1
.
5
1
2
4
.
3
1
0
.
5
1
2
2
1
.
5
4
100
200
300
400
500
Mass-to-Charge (m/z)
7
x10
1
0.9
0.8
0.7
0.6
0.5
0.4
0.3
0.2
0.1
0
1
.
7
0
2
0
.
1
0
9
.
1
3
1
.
3
6
0
0
1
9
1
.
.
7
1
.
5
6
4
4
2
3
100
200
300
400
Mass-to-Charge (m/z)
6
x10
6.5
6
5.5
5
4.5
4
3.5
3
2.5
2
1.5
1
0.5
0
1
.
1
8
2
1
.
0
5
.
1
3
6
.
0
1
3
9
2
.
1
.
3
1
3
3
.
1
0
8
7
1
2
100
200
300
400
Mass-to-Charge (m/z)
6
x10
1.4
1.3
1.2
1.1
1
0.9
0.8
0.7
0.6
0.5
0.4
0.3
0.2
0.1
0
1
.
5
5
3
0
.
7
6
2
1
.
3
7
0
.
1
0
1
.
5
.
1
3
9
2
.
1
9
3
4
.
1
3
5
5
4
1
100
200
300
400
Mass-to-Charge (m/z)
6.07 min
7.77 min
8.64 min
9.56 min
10.39 min
10.90 min

## Slide 5
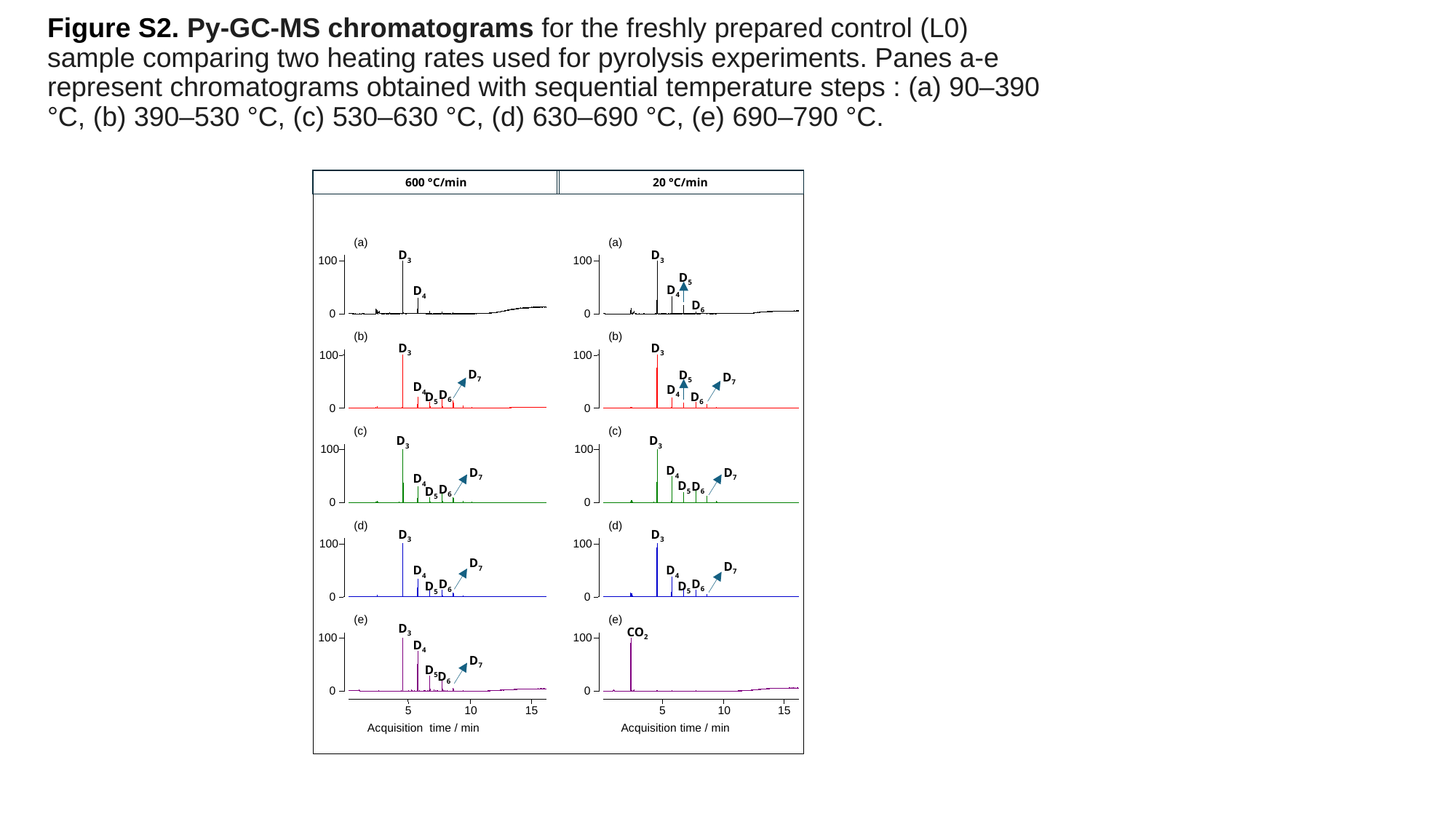

# Figure S2. Py-GC-MS chromatograms for the freshly prepared control (L0) sample comparing two heating rates used for pyrolysis experiments. Panes a-e represent chromatograms obtained with sequential temperature steps : (a) 90–390 °C, (b) 390–530 °C, (c) 530–630 °C, (d) 630–690 °C, (e) 690–790 °C.
600 °C/min
20 °C/min
(a)
100
0
(b)
100
0
(c)
100
0
(d)
100
0
(e)
100
0
5
10
15
(a)
100
0
(b)
100
0
(c)
100
0
(d)
100
0
(e)
100
0
5
10
15
Acquisition time / min
Acquisition time / min
D3
D3
D5
D4
D4
D6
D3
D3
D7
D5
D7
D4
D4
D6
D5
D6
D3
D3
D4
D7
D7
D4
D5
D6
D6
D5
D3
D3
D7
D7
D4
D4
D6
D6
D5
D5
D3
CO2
D4
D7
D5
D6

## Slide 6
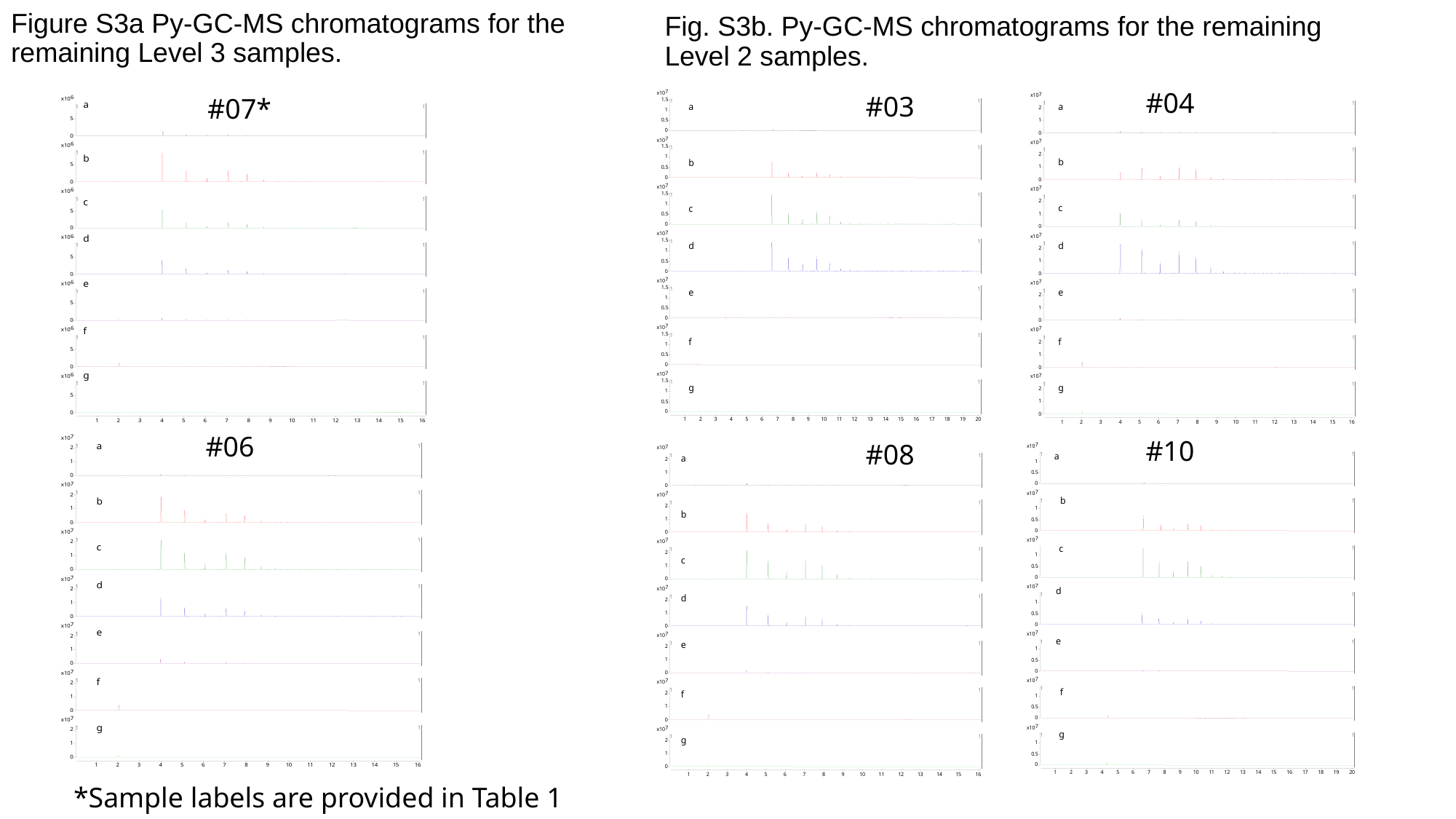

Figure S3a Py-GC-MS chromatograms for the remaining Level 3 samples.
Fig. S3b. Py-GC-MS chromatograms for the remaining Level 2 samples.
#04
#03
#10
#08
#07*
#06
*Sample labels are provided in Table 1

## Slide 7
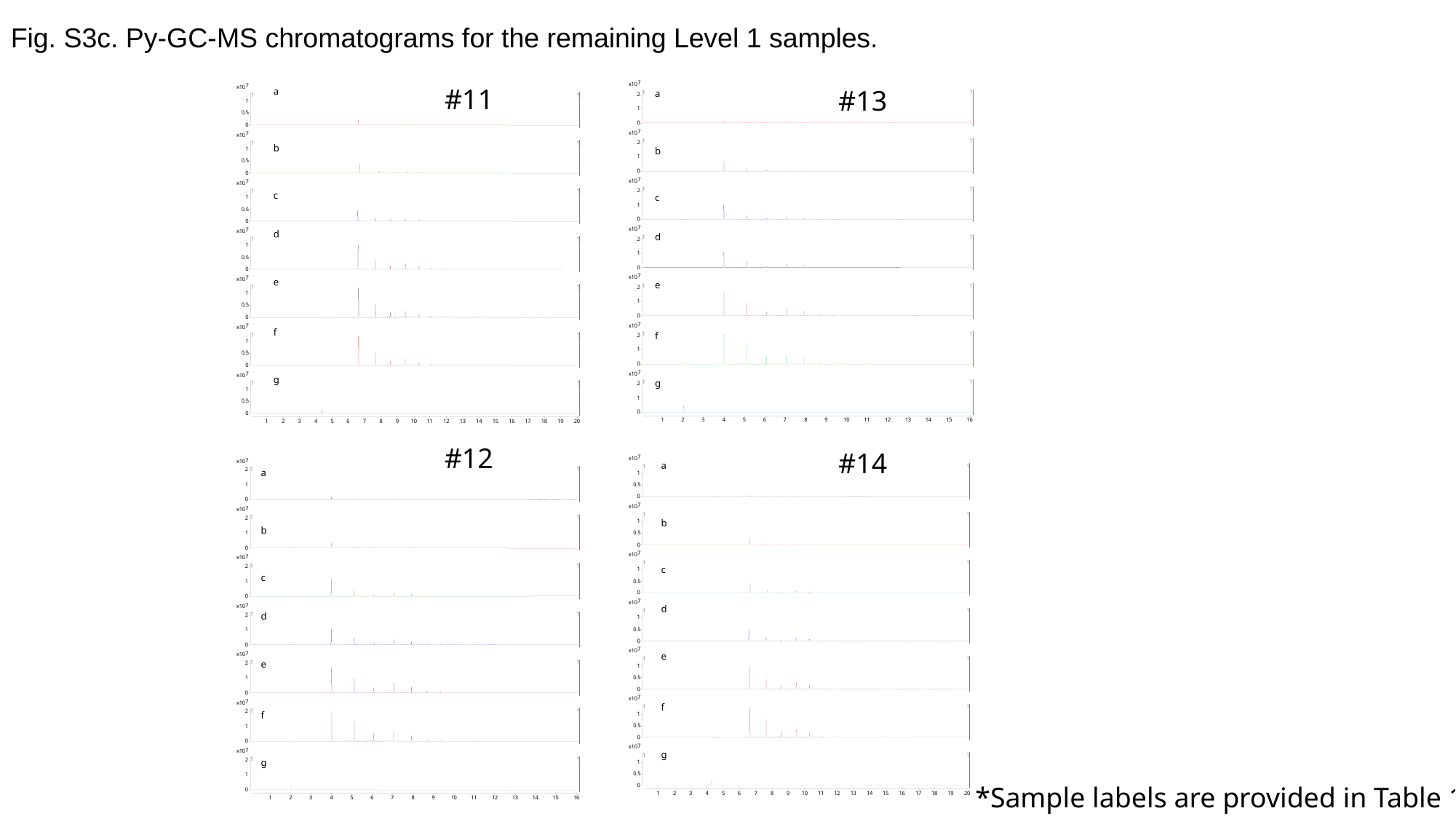

Fig. S3c. Py-GC-MS chromatograms for the remaining Level 1 samples.
#11
#13
#12
#14
*Sample labels are provided in Table 1

## Slide 8
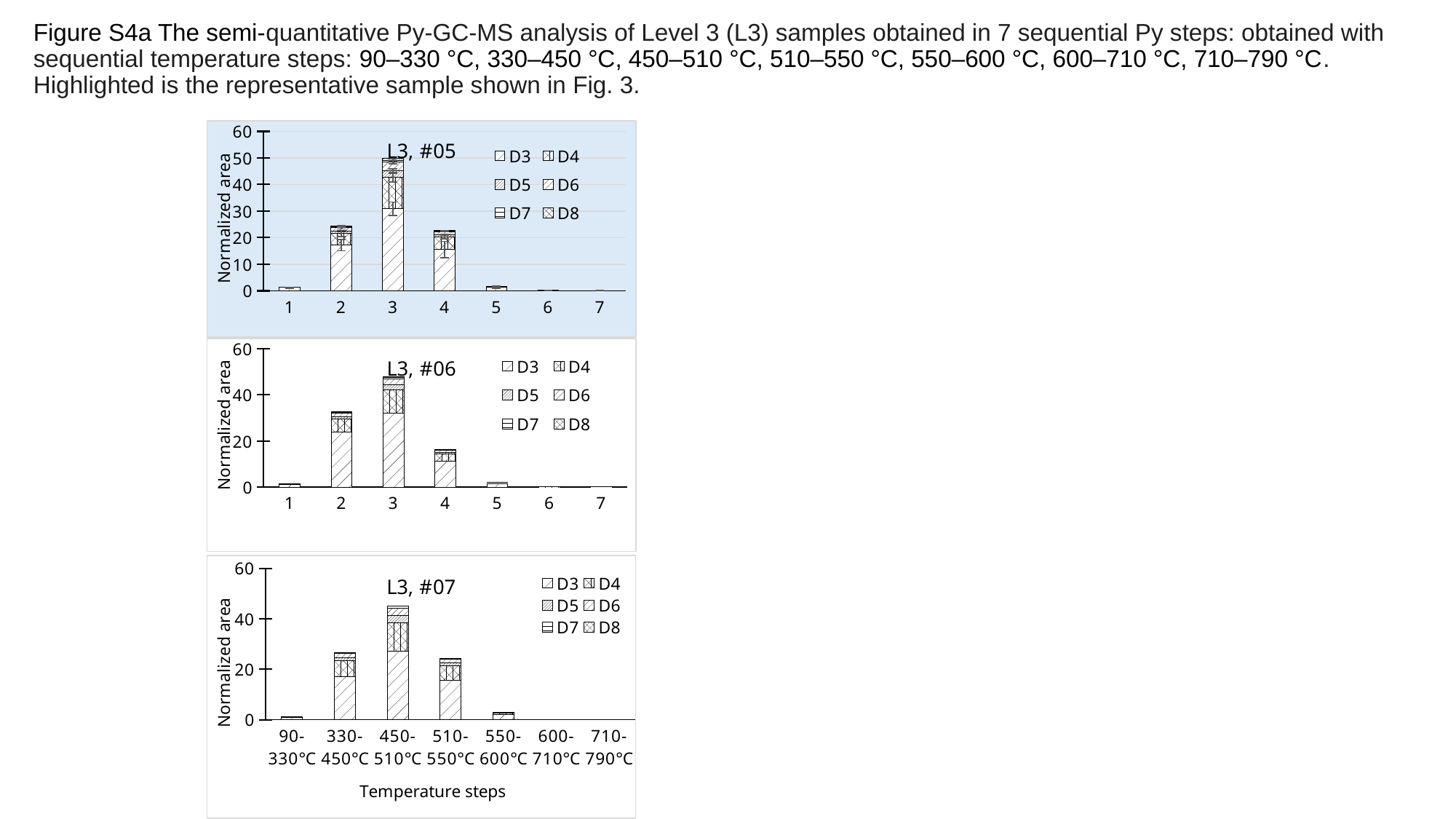

# Figure S4a The semi-quantitative Py-GC-MS analysis of Level 3 (L3) samples obtained in 7 sequential Py steps: obtained with sequential temperature steps: 90–330 °C, 330–450 °C, 450–510 °C, 510–550 °C, 550–600 °C, 600–710 °C, 710–790 °C. Highlighted is the representative sample shown in Fig. 3.
### Chart: L3, #05
| Category | D3 | D4 | D5 | D6 | D7 | D8 |
|---|---|---|---|---|---|---|
### Chart: L3, #06
| Category | D3 | D4 | D5 | D6 | D7 | D8 |
|---|---|---|---|---|---|---|
### Chart: L3, #07
| Category | D3 | D4 | D5 | D6 | D7 | D8 |
|---|---|---|---|---|---|---|
| 90-330°C | 0.9427991497536227 | 0.19466280064709418 | 0.05289566236106759 | 0.031206465826625028 | 0.0033914682524181783 | 0.0 |
| 330-450°C | 17.123985356753124 | 6.1319195066495435 | 1.2499069997196146 | 1.7226164308564904 | 0.4101935790740717 | 0.005131692236781249 |
| 450-510°C | 27.021549023189745 | 11.4927380721464 | 2.849980135304123 | 2.928603851394754 | 0.7582149275051138 | 0.013018591055443654 |
| 510-550°C | 15.537121629434411 | 5.872607782541497 | 1.1819517523323408 | 1.2610648845490207 | 0.2558389532477134 | 0.003070214634463983 |
| 550-600°C | 2.1090665814475784 | 0.5637907680942477 | 0.08481000911440957 | 0.06272763421113656 | 0.009413174119366442 | 8.427424638292704e-05 |
| 600-710°C | 0.05801554843836993 | 0.02166696562594551 | 0.0029965153994880095 | 0.0013716055578550624 | 0.0 | 0.0 |
| 710-790°C | 0.031820896776371926 | 0.00810138860871611 | 0.0010420573696625734 | 0.0006236515249925835 | 0.0 | 0.0 |

## Slide 9
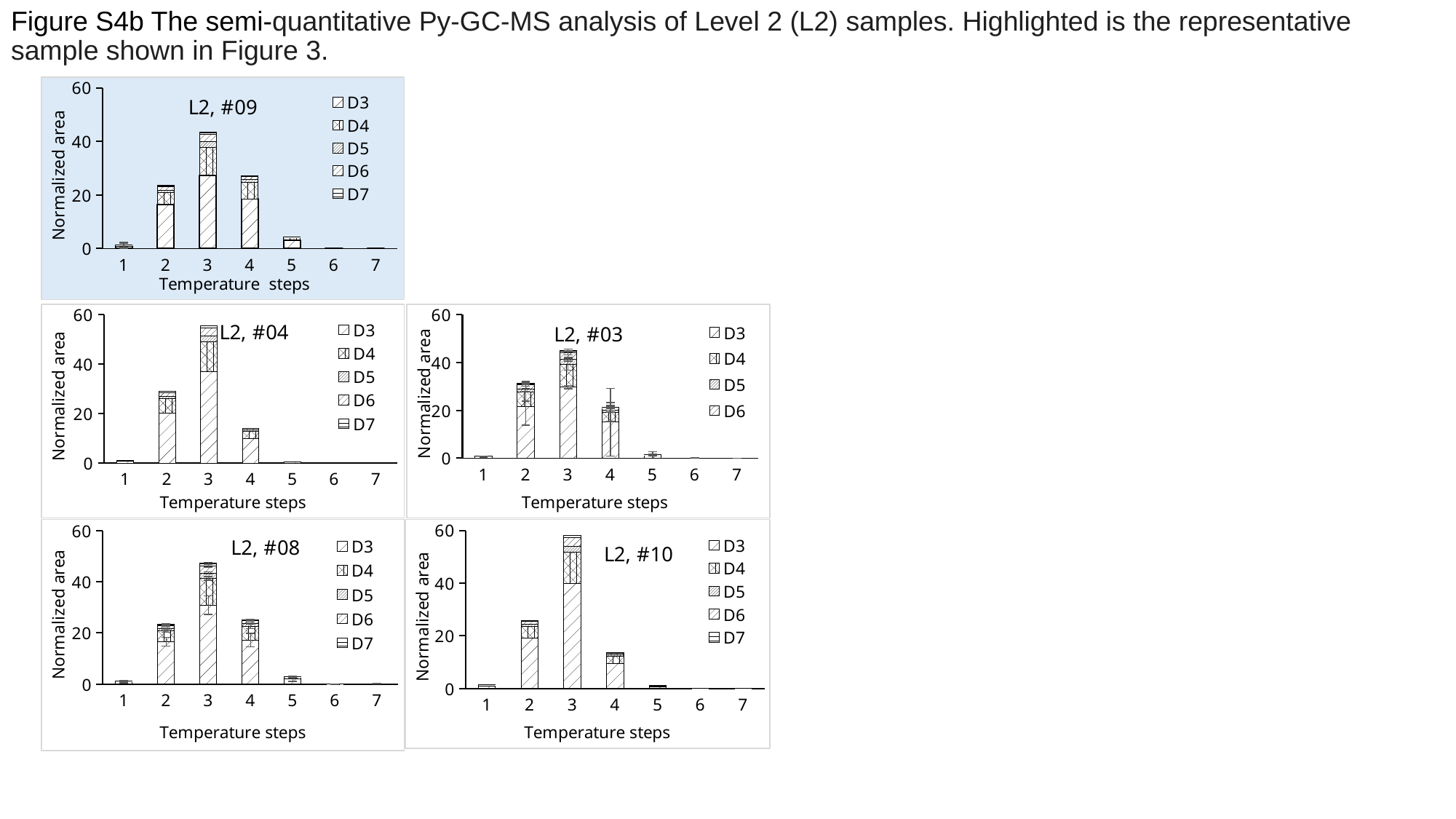

# Figure S4b The semi-quantitative Py-GC-MS analysis of Level 2 (L2) samples. Highlighted is the representative sample shown in Figure 3.
### Chart: L2, #09
| Category | D3 | D4 | D5 | D6 | D7 | D8 |
|---|---|---|---|---|---|---|
### Chart: L2, #04
| Category | D3 | D4 | D5 | D6 | D7 | D8 |
|---|---|---|---|---|---|---|
### Chart: L2, #03
| Category | D3 | D4 | D5 | D6 | D7 | D8 |
|---|---|---|---|---|---|---|
### Chart: L2, #08
| Category | D3 | D4 | D5 | D6 | D7 | D8 |
|---|---|---|---|---|---|---|
### Chart: L2, #10
| Category | D3 | D4 | D5 | D6 | D7 | D8 |
|---|---|---|---|---|---|---|

## Slide 10
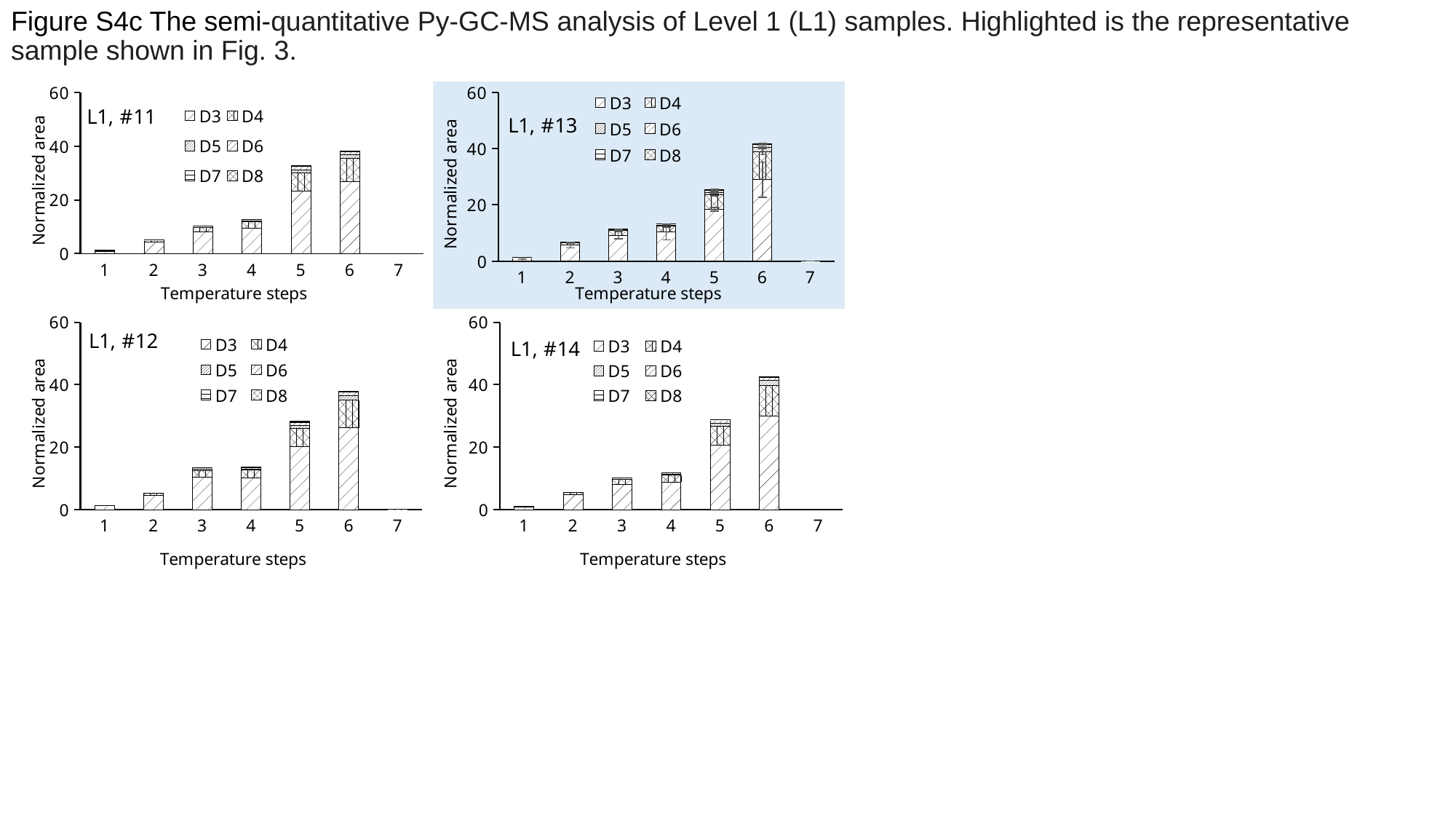

# Figure S4c The semi-quantitative Py-GC-MS analysis of Level 1 (L1) samples. Highlighted is the representative sample shown in Fig. 3.
### Chart: L1, #13
| Category | D3 | D4 | D5 | D6 | D7 | D8 |
|---|---|---|---|---|---|---|
### Chart: L1, #11
| Category | D3 | D4 | D5 | D6 | D7 | D8 |
|---|---|---|---|---|---|---|
### Chart: L1, #14
| Category | D3 | D4 | D5 | D6 | D7 | D8 |
|---|---|---|---|---|---|---|
### Chart: L1, #12
| Category | D3 | D4 | D5 | D6 | D7 | D8 |
|---|---|---|---|---|---|---|

## Slide 11
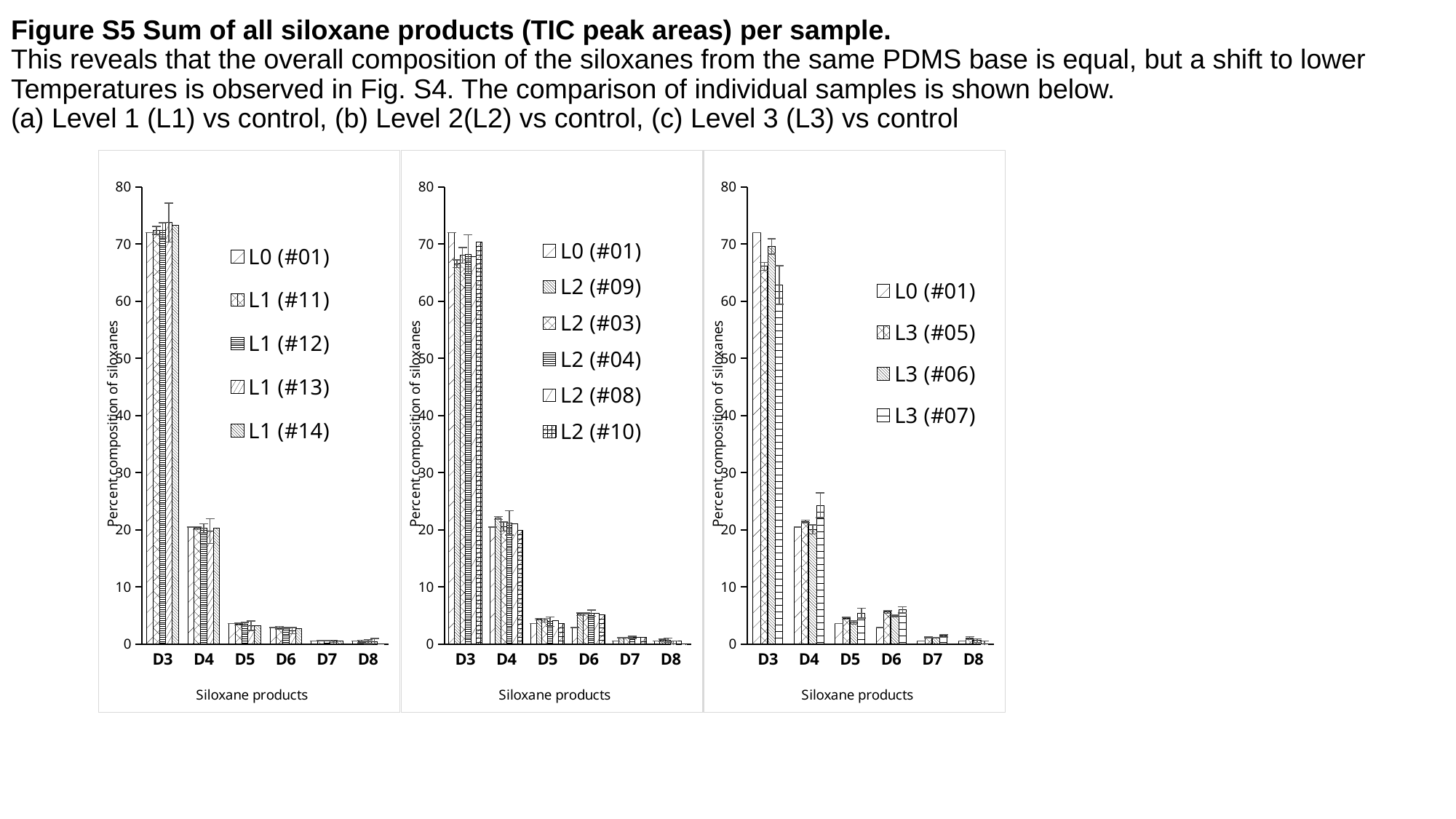

# Figure S5 Sum of all siloxane products (TIC peak areas) per sample. This reveals that the overall composition of the siloxanes from the same PDMS base is equal, but a shift to lower Temperatures is observed in Fig. S4. The comparison of individual samples is shown below. (a) Level 1 (L1) vs control, (b) Level 2(L2) vs control, (c) Level 3 (L3) vs control
### Chart
| Category | L0 (#01) | L1 (#11) | L1 (#12) | L1 (#13) | L1 (#14) |
|---|---|---|---|---|---|
| D3 | 71.98695744445672 | 72.36149257653247 | 72.36149257653247 | 73.7534560029331 | 73.25913525317588 |
| D4 | 20.472408349528067 | 20.261575959580718 | 20.261575959580718 | 19.782430235486533 | 20.243389757544616 |
| D5 | 3.5983842459938074 | 3.4881192750191232 | 3.4881192750191232 | 3.204575455477495 | 3.244590947493915 |
| D6 | 2.8698391175040365 | 2.863975606329499 | 2.863975606329499 | 2.365034020256504 | 2.7064002117524826 |
| D7 | 0.5563883252404364 | 0.589614391736914 | 0.589614391736914 | 0.4746290181417315 | 0.5364635034272479 |
| D8 | 0.516022517276938 | 0.4352221908012856 | 0.4352221908012856 | 0.4198752677046414 | 0.010020326605847727 |
### Chart
| Category | L0 (#01) | L2 (#09) | L2 (#03) | L2 (#04) | L2 (#08) | L2 (#10) |
|---|---|---|---|---|---|---|
| D3 | 71.98695744445672 | 66.54998228520707 | 68.01247029053219 | 68.20844931567719 | 67.7578010917399 | 70.30274470371468 |
| D4 | 20.472408349528067 | 22.077988733521682 | 20.575864190723205 | 21.222240796848414 | 21.0907405705669 | 19.871520301847962 |
| D5 | 3.5983842459938074 | 4.361880894222569 | 4.176516709401611 | 3.9208227331566974 | 4.102940883595323 | 3.553181459103281 |
| D6 | 2.8698391175040365 | 5.239009254964069 | 5.354116643051856 | 5.429308430354572 | 5.34922403673831 | 5.130964044350917 |
| D7 | 0.5563883252404364 | 1.135144149329869 | 1.1622842917091176 | 1.1983497237104084 | 1.2004053023950443 | 1.124522017464223 |
| D8 | 0.516022517276938 | 0.6359946827547488 | 0.7187478745820272 | 0.02082900025271235 | 0.4988881149645081 | 0.017067473518930117 |
### Chart
| Category | L0 (#01) | L3 (#05) | L3 (#06) | L3 (#07) |
|---|---|---|---|---|
| D3 | 71.98695744445672 | 66.09206181131658 | 69.59576476207356 | 62.82435818579322 |
| D4 | 20.472408349528067 | 21.45378766753379 | 20.062663858733888 | 24.285487284313447 |
| D5 | 3.5983842459938074 | 4.550201052778232 | 3.7609165814129137 | 5.423583131600707 |
| D6 | 2.8698391175040365 | 5.607074142745802 | 4.916050147130641 | 6.008214523920876 |
| D7 | 0.5563883252404364 | 1.1920733825056107 | 1.0602255993763503 | 1.4370521021986837 |
| D8 | 0.516022517276938 | 1.1048019431199936 | 0.6043790512726605 | 0.021304772173071817 |

## Slide 12
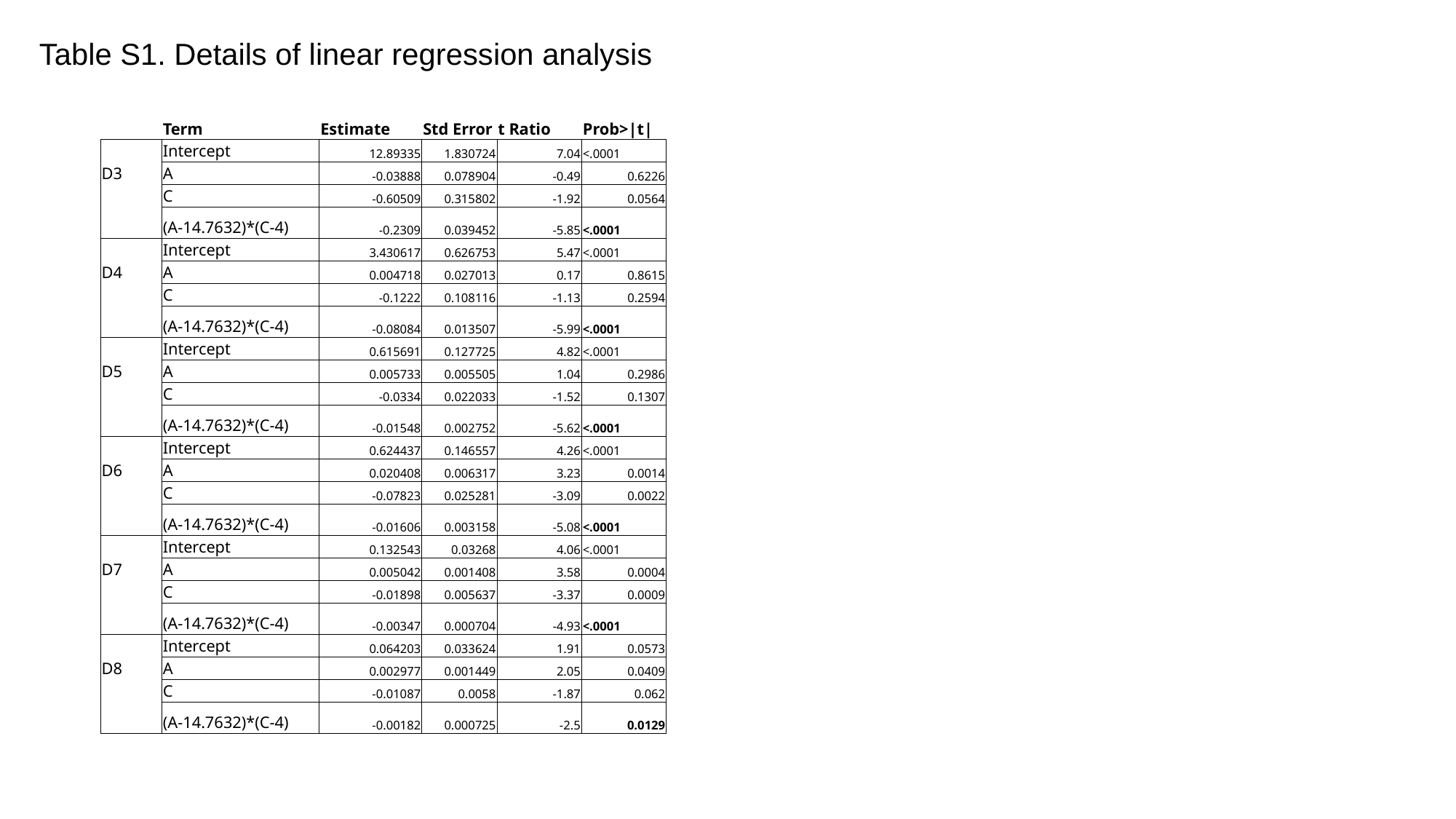

# Table S1. Details of linear regression analysis
| | Term | Estimate | Std Error | t Ratio | Prob>|t| |
| --- | --- | --- | --- | --- | --- |
| | Intercept | 12.89335 | 1.830724 | 7.04 | <.0001 |
| D3 | A | -0.03888 | 0.078904 | -0.49 | 0.6226 |
| | C | -0.60509 | 0.315802 | -1.92 | 0.0564 |
| | (A-14.7632)\*(C-4) | -0.2309 | 0.039452 | -5.85 | <.0001 |
| | Intercept | 3.430617 | 0.626753 | 5.47 | <.0001 |
| D4 | A | 0.004718 | 0.027013 | 0.17 | 0.8615 |
| | C | -0.1222 | 0.108116 | -1.13 | 0.2594 |
| | (A-14.7632)\*(C-4) | -0.08084 | 0.013507 | -5.99 | <.0001 |
| | Intercept | 0.615691 | 0.127725 | 4.82 | <.0001 |
| D5 | A | 0.005733 | 0.005505 | 1.04 | 0.2986 |
| | C | -0.0334 | 0.022033 | -1.52 | 0.1307 |
| | (A-14.7632)\*(C-4) | -0.01548 | 0.002752 | -5.62 | <.0001 |
| | Intercept | 0.624437 | 0.146557 | 4.26 | <.0001 |
| D6 | A | 0.020408 | 0.006317 | 3.23 | 0.0014 |
| | C | -0.07823 | 0.025281 | -3.09 | 0.0022 |
| | (A-14.7632)\*(C-4) | -0.01606 | 0.003158 | -5.08 | <.0001 |
| | Intercept | 0.132543 | 0.03268 | 4.06 | <.0001 |
| D7 | A | 0.005042 | 0.001408 | 3.58 | 0.0004 |
| | C | -0.01898 | 0.005637 | -3.37 | 0.0009 |
| | (A-14.7632)\*(C-4) | -0.00347 | 0.000704 | -4.93 | <.0001 |
| | Intercept | 0.064203 | 0.033624 | 1.91 | 0.0573 |
| D8 | A | 0.002977 | 0.001449 | 2.05 | 0.0409 |
| | C | -0.01087 | 0.0058 | -1.87 | 0.062 |
| | (A-14.7632)\*(C-4) | -0.00182 | 0.000725 | -2.5 | 0.0129 |
